# Transactions American Medical Association

**Published:** 1865-10

**Authors:** 


### Transactions American Medical Association.

PHILADELPHIA, Sept. 28, 1865.

Dear Doctor:—Just received your Journal for September. By the enclosed Circular you will see that the subscription price is ($5) five dollars. Therefore you will please, in your next issue, change 3 to 5. The volume is fast approaching completion, and I hope shortly to place the copies in the hands of the subscribers.

I shall be most happy at any time to furnish any information relative to the Association to yourself or subscribers, and also would be glad of any suggestions for the benefit of that body, which, if properly sustained, is destined to become a vast power in the profession.

Very truly yours, etc. WM. B. ATKINSON.

PHILADELPHIA, September, 1865.

Dear Sir:—In accordance with a resolution of the AMERICAN MEDICAL ASSOCIATION adopted at the meeting held in Boston, June last, you are respectfully invited to contribute the sum of *Two Dollars*, in addition to your assessment, to meet the expense of printing the volume of Transactions of that meeting.

The volume will make about 842 pages at an estimated cost of $3,400. There are in the hands of the Treasurer only 2,200, leaving a deficit of $1,200 to be made up by subscription, and without which the volume cannot be issued. The high price of labor and material, and the large number of illustrations make it impossible to reduce the estimate below the amount named, and the Committee of Publication are therefore reluctantly compelled to make this appeal in order to enable them to go on with the work.

Please encolse your contribution to Dr. CASPAR WISTER, No. 1303 Arch Street, Philadelphia.

Very respectfully, yours,
FRANCIS G. SMITH, JR.,
*Chairman Com. of Publication.*

